# Epidemiology of SARS-CoV-2 Infection in Italy Using Real-World Data: Methodology and Cohort Description of the Second Phase of Web-Based EPICOVID19 Study

**DOI:** 10.3390/ijerph19031274

**Published:** 2022-01-24

**Authors:** Fulvio Adorni, Nithiya Jesuthasan, Elena Perdixi, Aleksandra Sojic, Andrea Giacomelli, Marianna Noale, Caterina Trevisan, Michela Franchini, Stefania Pieroni, Liliana Cori, Claudio Maria Mastroianni, Fabrizio Bianchi, Raffaele Antonelli-Incalzi, Stefania Maggi, Massimo Galli, Federica Prinelli, on behalf of the EPICOVID19 Working Group

**Affiliations:** 1 National Research Council, Institute of Biomedical Technologies, Via Fratelli Cervi 93, 20054 Segrate, Italy; nithiya.jesuthasan@itb.cnr.it (N.J.); elenaperdixi@gmail.com (E.P.); aleksandra.sojic@itb.cnr.it (A.S.); federica.prinelli@itb.cnr.it (F.P.); 2 Infectious Diseases Unit, Department of Biomedical and Clinical Sciences L. Sacco, Università di Milano, ASST Fatebenefratelli Sacco, 20157 Milan, Italy; dott.giacomelli@gmail.com (A.G.); massimo.galli@unimi.it (M.G.); 3 National Research Council, Neuroscience Institute, Aging Branch, Via Vincenzo Maria Gallucci 16, 35128 Padova, Italy; marianna.noale@in.cnr.it (M.N.); stefania.maggi@in.cnr.it (S.M.); 4 Geriatric Unit, Department of Medicine (DIMED), University of Padova, Via Giustiniani 2, 35128 Padova, Italy; caterina.trevisan.5@studenti.unipd.it; 5 Department of Medical Sciences, University of Ferrara, Via Aldo Moro 8, Cona, 44124 Ferrara, Italy; 6 National Research Council, Institute of Clinical Physiology, Via G. Moruzzi 1, 56124 Pisa, Italy; michela.franchini@ifc.cnr.it (M.F.); stefania.pieroni@ifc.cnr.it (S.P.); liliana.cori@ifc.cnr.it (L.C.); fabriepi@ifc.cnr.it (F.B.); 7 Department of Public Health and Infectious Diseases, Sapienza University of Rome, 00185 Rome, Italy; claudio.mastroianni@uniroma1.it; 8 Unit of Geriatrics, Department of Medicine, Biomedical Campus of Rome, 00128 Rome, Italy; r.antonelli@unicampus.it

**Keywords:** SARS-CoV-2, COVID-19, testing, observational study, web-based survey, self-reported data, public health

## Abstract

Digital technologies have been extensively employed in response to the SARS-CoV-2 pandemic worldwide. This study describes the methodology of the two-phase internet-based EPICOVID19 survey, and the characteristics of the adult volunteer respondents who lived in Italy during the first (April–May 2020) and the second wave (January–February 2021) of the epidemic. Validated scales and ad hoc questionnaires were used to collect socio-demographic, medical and behavioural characteristics, as well as information on COVID-19. Among those who provided email addresses during phase I (105,355), 41,473 participated in phase II (mean age 50.7 years ± 13.5 SD, 60.6% females). After a median follow-up of ten months, 52.8% had undergone nasopharyngeal swab (NPS) testing and 13.2% had a positive result. More than 40% had undergone serological test (ST) and 11.9% were positive. Out of the 2073 participants with at least one positive ST, 72.8% had only negative results from NPS or never performed it. These results indicate that a large fraction of individuals remained undiagnosed, possibly contributing to the spread of the virus in the community. Participatory online surveys offer a unique opportunity to collect relevant data at individual level from large samples during confinement.

## 1. Introduction

The coronavirus disease 2019 (COVID-19) caused by the severe acute respiratory syndrome coronavirus-2 (SARS-CoV-2), has posed an unprecedented public health emergency worldwide [1]. From the disease outbreak in February 2020 to 20 December 2021, with 5,389,155 confirmed cases and 135,641 deaths, Italy was the first Western country to be severely affected by the COVID-19 pandemic [2].

During the first wave of the pandemic peak worldwide, epidemiological surveillance strategies were mainly based on the testing of symptomatic patients with serious diseases requiring hospitalization and intensive medical care [3,4]. Despite efforts to ensure universal access to molecular testing, the massive spread of the infection has de facto restricted the diagnosis of COVID-19 only to infected people who exhibited severe symptoms. This limitation, combined with the lack of official standards in the detection and diagnosis of asymptomatic or pauci-symptomatic patients, heavily affected the effectiveness of testing strategies and contact tracing, which in turn compromised the control of the spread of SARS-CoV-2 in the community [5]. As a result of the limited availability of population-based data, the inconsistency between official statistics of different countries has made a global comparison difficult [6].

To easily and freely collect real-time and population-based data, multiple eHealth technologies have been employed [7]. In several countries, such as the UK [8], US [9], Israel [10], and Canada [11,12], large numbers of participants were recruited via mobile applications and web-based tools, to collect information on symptoms, psychosocial determinants, behavioural changes; to monitor positive cases; and in some circumstances to carry out contact tracing.

The results of participatory surveillance platforms have contributed to increasing knowledge of the characteristics of SARS-CoV-2 infection and associated factors at the population level, especially in areas with insufficient testing capacity. Typical symptom patterns like anosmia, dysgeusia, fever, shortness of breath, and cough were consistently observed in association with the self-reported positive SARS-CoV-2 test, highlighting the relevance of collaborative syndromic surveillance during pandemic waves worldwide [13]. Furthermore, digital epidemiological surveillance has filled the gaps due to the lack of seroprevalence studies, attempting to size up more completely the real, yet unknown, spread of the epidemic.

In response to the COVID-19 pandemic and to the lack of Italian epidemiological data on persons who experienced the mild-to-severe disease in the general population, a large sample of more than 198,000 voluntary adults who lived in Italy during the first lockdown was recruited through a web-based approach. These data allowed to better understand the association of symptoms (or cluster of symptoms) [14,15,16] and smoking habits [17] with COVID-19, the role of vaccination for other vaccine-preventable diseases [18,19], as well as to characterize psychological aspects of the population [20] and health policy issues [21] in the context of the pandemic. During the second wave of the epidemic in Italy, a follow-up questionnaire was sent by e-mail to collect further data on SARS-CoV-2 testing, COVID-19 related symptoms, hospitalization, and behavioural and psychosocial factors associated with the pandemic. This article describes the rationale, methodology, and socio-demographic and clinical characteristics of people who participated in the second phase of the internet-based EPICOVID19 study in Italy, in January–February 2021.

## 2. Materials and Methods

### 2.1. Development of the EPICOVID19 Questionnaires

EPICOVID19 is an Italian national internet-based survey with a cross-sectional research design in phase I [14] and a longitudinal design in phase II, carried out on a self-selected sample of adult volunteers (18+ years old) living in Italy during the first and second waves of the pandemic. Study design and data were registered in ClinicalTrials.gov (https://clinicaltrials.gov/ct2/show/NCT04471701, accessed on 18 December 2021). The EPICOVID19 study was established as a collaborative project of a working group including epidemiologists, physicians with expertise in infectious diseases, biostatisticians, and public health professionals, with the aim of improving knowledge about SARS-CoV-2 infection. The EPICOVID19 survey was designed after a comprehensive literature review of existing research as to ensure maximal harmonization and comparability with other large population studies. Most of the items in the questionnaire were chosen based on standardized, validated scales. We checked the clarity of the items before launching the questionnaire to the general population in order to avoid as much as possible misunderstandings in the questions and answers and refine readability. With this aim and for both phase I and phase II, we asked a group of 20 volunteers from outside the working team, chosen by convenience, aged between 18 and 70 years, sex-balanced, and with different levels of education, to fill out the questionnaire and to provide us with their feedback on its compilation. Following the feedback received, we finalized the questionnaires after adjusting the question flow and improving the simplicity of the language.

### 2.2. Content of the EPICOVID19 Questionnaire

Participants were asked to complete the two questionnaires (phase I and II) after reading an introductory page (which briefly described the rationale and objectives of the study and the scientific consortium), and after accepting the option to provide consent to participate. The content of the first questionnaire was described in a previous publication [14]. The phase II questionnaire is included in Annex 1, and a comparison of its content with the one of phase I is presented in Table S1. The validated scales and questionnaires used in the two surveys are described in Table S2.

### 2.3. Sample Recruitment and Study Population

**Figure 1:**
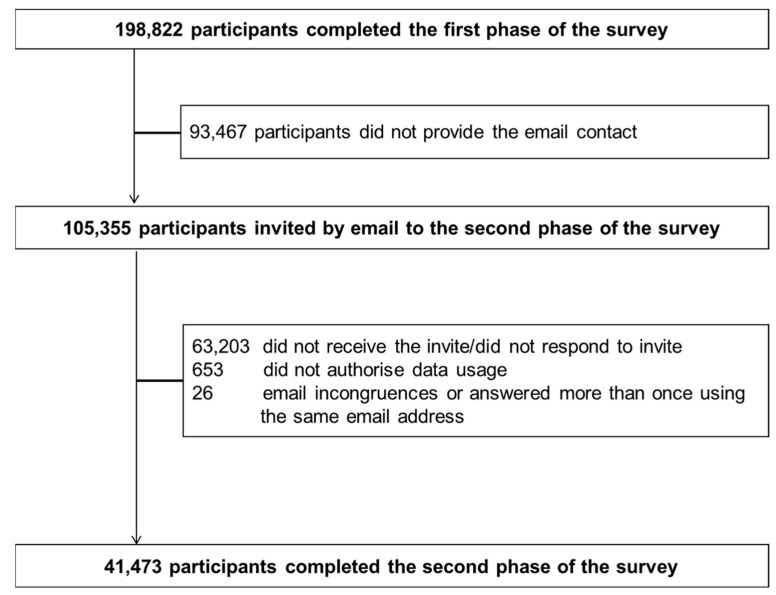
Flow-chart of the participants in phase II EPICOVID19 survey.

The two-wave web-based surveys were implemented using the European Commission’s official open-source management tool EUSurvey (https://ec.europa.eu/eusurvey, accessed on 18 December 2021). The link to the first questionnaire was shared since 13 April to 2 June 2020, when the Italian government was applying the strictest lockdown on the entire population. Participation was asked through mailing lists, social media platforms (Facebook, Twitter, Instagram, WhatsApp), press releases, internet pages, television and radio news programmes, word of mouth and the study website (https://epicovid19.itb.cnr.it/, accessed on 18 December 2021). Inclusion criteria were age ≥ 18 years, access to a mobile phone, computer, or tablet with internet connectivity and provision of online consent to participate in the study. In total, 207,341 participants clicked on the first questionnaire link and 198,822 provided consent to participate and completed the first online survey. Participants who had consented to be contacted (*n* = 105,355, 53%), by providing their personal email address during the first survey, received an email invitation (from 15 January to 28 February 2021) containing a personalized link that allowed them to complete the second questionnaire. In that period, the restrictions in Italy were less severe than during the first phase of the survey. Those who had not completed the EPICOVID19 phase II questionnaire within fifteen days since the invitation received up to three reminder emails. Exclusion of participants who did not receive the invitation or did not respond (*n* = 63,203), who did not provide consent (*n* = 653), and of those with inconsistencies in email contacts or who answered more than once using the same email address (*n* = 26) resulted in 41,473 respondents included in the present analysis (Figure 1). Excluded participants (*n* = 157,349) were younger, more likely residents in Southern regions or islands, with a lower educational level, and more frequently students (Table S3).

### 2.4. Variables Collected and Data Transformations

Variables of interest for the present study were the following: socio-demographic information (age, education, employment, job position at-risk for the infection, socio-economic status), body mass index (BMI, calculated as weight divided by height squared), number of chronic diseases (listed in Annex 1, question #13), smoking habit, alcohol consumption, self-perceived health status [22] recoded as bad or very bad, adequate, and good or very good. Townsend Deprivation Scores (TDSs) was calculated as a proxy for individual level deprivation [23] by summing up, for each participant, the following variables (both dichotomized): unemployment, non-ownership of the house where he/she lives, no car owned by family members, and house crowding (defined as number of cohabitants greater than the number of rooms in the house, kitchen and bathrooms excluded). The total score ranged from 0 to 4, with higher scores indicating higher deprivation. Sleep problems were measured using the Jenkins Sleep Scale (JSS) [24] based on four items. Each one was rated on a Likert-like scale from 0 to 5, and the total score was the sum of all four items’ scores and ranged from 0 (no sleep problems) to 20. The continuous score was dichotomized as follows: score lower than 12 showing a low frequency of sleep disturbances and score greater than 11 indicating high frequency of sleep disturbances) [25]. Personal stress was measured using the 10-item Perceived Stress Scale (PSS) [26] and adding five items developed ad hoc. Each item was rated on a Likert-like scale of 0 to 4. The score was obtained firstly by reversing responses (0 = 4, 1 = 3, 2 = 2, 3 = 1, and 4 = 0) to the four positively stated items (items 4, 5, 7, and 8) and then summing across all scale items. Individual scores fell in the range 0–40, higher scores indicating higher perceived stress. The score was categorized as follows: 0–13: low stress; 14–26: moderate stress; 27–40 high stress. Fear of contagion for oneself or relatives, fear about personal economic and job situation, and fear about the relatives’ economic and job situation were assessed with a short questionnaire developed ad hoc for the present survey. Each aspect was rated on a Likert-like scale from 0 (no fear) to 4, and the total score was the sum of all four items’ scores and ranged from 0 to 16, with higher scores indicating higher fear. Individual feelings about being sufficiently informed about COVID-19 was dichotomized into a binary variable.

COVID-19-related variables have been reported including: contacts with COVID-19 cases, self-isolation, nasopharyngeal swab test (NPS) (numbers, results, reasons for having performed the positive test, places attended before the positive test), hospitalization, serological test (ST) (results, reasons for having performed the positive test), anti-COVID-19 vaccination(s), and SARS-CoV-2 infection-related symptoms.

### 2.5. Statistical Analysis

The continuous variables were represented as mean and standard deviation (SD) and the categorical variables were expressed as numbers and percentages. The Student t-test and Chi-square test were used to compare the respondents’ characteristics by sex for continuous and categorical variables, respectively. The threshold of statistical significance for any test was set at *p*-values of 0.05. All of the statistical analyses were carried out using STATA software packages (version 15, StataCorp LP, 347 College Station, TX, USA) and SPSS (IBM Corp. Released, IBM SPSS Statistics version 25.0 Armonk, NY: IBM Corp.). The response rate map was drawn using the open-source data visualization Datawrapper GmbH tool (https://app.datawrapper.de/signin, accessed on 19 October 2021).

### 2.6. Dissemination and Provision of Results to Participants

The results of the first phase of the EPICOVID19 web-based survey were communicated mainly through peer-reviewed publications [14,15,16,17,18,19,20,21] in international scientific journals, meetings and conference presentations, workshops, the study website (www.epicovid19.itb.cnr.it, accessed on 18 December 2021), and disseminated through audio and video interviews and local printed media. A personalized e-mail with the provision of the results was sent to each participant who completed the survey and accepted to be contacted for communications about the project.

## 3. Results

**Figure 2:**
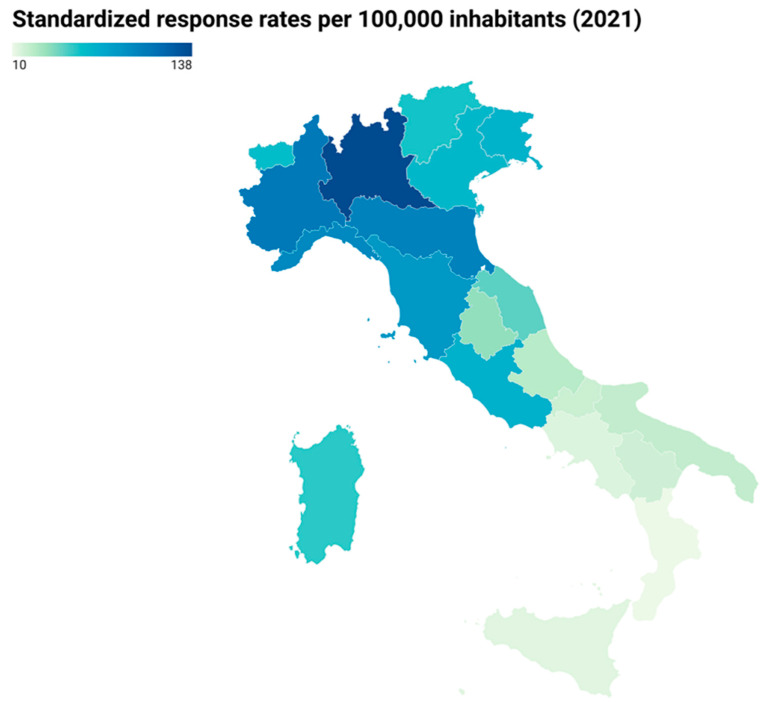
Response rates by Italian region in the phase II survey. Darker coloured regions indicate higher response rates.

The standardized response rates per 100,000 inhabitants by Italian regions over the January–February 2021 study period are represented in Figure 2 and Table S4. The percentages relating to the regional distribution of the Italian population were taken from the ISTAT website [27]. Darker coloured regions in Figure 2 indicate higher response rates which were mostly in northern Italy (Lombardia 137.5, Piemonte 106.6, Emilia-Romagna 100.6).

Table 1 summarizes the personal characteristics of the 41,473 participants who completed the phase II study according to sex. The mean age of the sample was 50.7 years ± 13.5 SD (females 49.8 ± 13.0; males 52.2 ± 14.3) and 65.5% (*n* = 27,158) had a university degree or post-graduate qualification. Respondents were mostly employed with stable positions (26,124, 63%); during the emergency period, 44.5% (12,277) and 37.9% (10,458) continued to work on-site and alternated work from home and on-site work, respectively. Relatively to the risk of infection, the most represented job categories were school staff (3653, 13.2%) and the healthcare workers (3523, 12.8%), with significant differences between males and females. A total of 0.6% had a high deprivation score (score ≥ 3). The mean BMI was 24.4 kg/m^2^ ± 3.9 SD (females 23.6 ± 4.0; males 25.6 ± 3.5) and 4.9% (*n* = 2044) of the whole sample reported three or more chronic diseases (5.2% females; 4.9% males). A total of 57.7% (*n* = 23,918) were never-smokers (59.4% among females and 55.0% among males) and 25,954 (62.6%: 68.2% females and 53.9% males) were teetotalers or consumed alcoholic beverages between meals less than 5 times a month. A percentage of 8.1% reported sleep disorders during the previous month and 78.7% (*n* = 32,630) rated their own health status as good or very good (77.4% females and 80.6% males). Most of the participants showed a low (50.9) or moderate (45.3) score at the PSS, with females having higher level of stress than males. More than 90% of the study participants felt they were sufficiently informed about the pandemic.

Table 2 reports COVID-19-related variables according to sex. Out of all the respondents, 70.6% (*n* = 29,300) never had close contact with COVID-19 cases and have never been in self-isolation (*n* = 29,275). More than half of the respondents (21,877, 52.8%) underwent molecular NPS testing, and among them 2902 (13.2%) tested positive at least once, with no differences between males (13.7%) and females (13.0%). One-fifth of tested participants performed more than four NPS during the study period and almost 90% underwent the molecular NPS test type instead of the rapid antigen-based test. The most frequent reason for the NPS testing with a positive result was the showing symptoms of COVID-19 (64.5%), followed by having contact with a COVID-19 case (44.2%); 41.3% referred to having shared the workplace within the 2 weeks before resulting positive to the NPS test. Among those who reported at least one positive NPS test, 359 (12.4%) were hospitalized, more frequently males (15.7%) than females (10.2%). During the study period, 41.9% of the respondents (*n* = 17,394) underwent ST, and among them 2073 (11.9%) tested positive at least once (11.8% females and 12.2% males). Half of the participants performed the test because of their own choice (49.9%). A total of 5.7% of the sample (*n* = 2371) received both doses of an anti-COVID-19 vaccine (6.5% females and 4.5% males).

**Figure 3:**
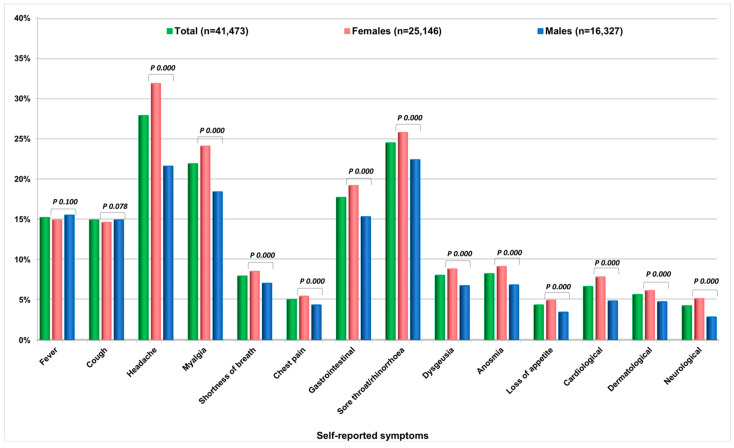
Frequency of self-reported symptoms by sex between March 2020 and February 2021 (*n* = 41,473) and *p*-values of the comparisons between females and males.

The three most frequent self-referred symptoms (Figure 3, Table S5) in the whole sample were headache (27.9%: 31.9% females and 21.6% males), sore throat/rhinorrhoea (24.5%: 25.6% females and 22.4% males), and myalgia (21.9%: 24.1% females and 18.4% males). Anosmia and dysgeusia were reported by 8.2% and 8.0% of the sample, and more frequently by females. Out of the 41,473 respondents of the second survey, 19,325 (46.6%) reported no symptoms (50.6% males and 44.0% females) (data not shown). On the other hand, among those with at least one positive NPS and/or ST (*n* = 4411), myalgia (64.6%: 67.6% females and 59.7% males), fever (58%: 55.4% females and 62.3% males), and headache (52.7%: 58.1% females and 44% males) were the three most frequent self-reported symptoms. Anosmia and dysgeusia accounted for 51.6% (females 55.9% and males 44.7%) and 48.3% (females 52.2% and males 42.0%), respectively.

**Figure 4:**
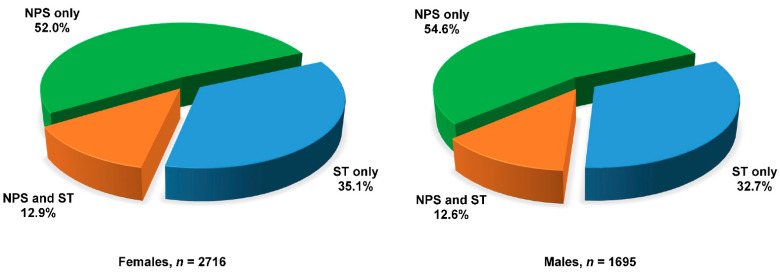
Distribution of positivity to SARS-CoV-2 by type of test performed in 4411 cases between March 2020 and February 2021 by sex. NPS: nasopharyngeal swab. ST: serological test.

During the period March 2020–February 2021, 33.3% (*n* = 13,805) did not perform any COVID-19 tests, 24.8% (*n* = 10,274) underwent NPS only, 14.0% (*n* = 5791) underwent ST only, whereas 28.0% (*n* = 11,603) performed both NPS and ST (data not shown). Out of the 2073 participants with at least one positive ST, 1509 (72.8%) had undergone one or more NPS always with negative results or never performed it. In the group of participants diagnosed (NPS or ST) with SARS-CoV-2 infection (*n* = 4411), more than one-third (Figure 4) was aware that they had the infection, which was not intercepted in its acute phase (NPS never executed or executed with negative result, before or after known seropositivity), with slight differences between sexes.

## 4. Discussion

This article provides a snapshot of the socio-demographic and clinical characteristics of the 41,473 respondents who participated in the second phase of the web-based EPICOVID19 study conducted in Italy during January–February 2021. It also shows the frequency of the NPS and/or ST tests, the prevalence of positivity to SARS-CoV-2 among the tested participants, and the frequency of COVID-19 related symptoms in a median study period of ten months since March 2020.

The EPICOVID19 questionnaires had the power to collect some data useful to characterize the individual behaviours of the respondents involving several aspects of daily life usually not collected in clinical context. The national coverage of the survey was in line with the geographical spread of COVID-19 during the first wave [2], when participants were recruited. As for work conditions, the majority of the participants (63%) maintained their stable work position with 18% shifting to work from home, the data in accordance with the Eurostat Statistics. In 2020, 12.3% of employed aged 15–64 years said they often work in agile mode in the European Union, and an identical percentage was reported in Italy (12.2%) [28]. Furthermore, according to the Smart Working Observatory of the Politecnico di Milano [29], during the lockdown about 6.6 million workers shifted to remote working. Among the work category at high risk of infection, school staff and healthcare workers represent almost 30% of the study sample, with a significant unbalance toward the female sex, as expected.

Regarding the perception of health status and mood disorders, 78.7% referred to perceive a good or very good health status with no substantial difference between females and males. However, 8.1% and 49.1% reported sleep disorders and moderate-to-high self-perceived stress during the month before the survey completion, respectively. Similar to these findings, recent studies reported a high prevalence of sleep problems [30] and a high level of stress or anxiety [31] during the COVID-19 outbreak. The results of the present study also showed that females are more likely to manifest sleep disorders and psychological stress as pointed out in other investigations [32,33]. Furthermore, females are more worried about contagion for themselves or relatives and about personal and relatives’ economic and job situation, confirming the results of phase I of the EPICOVID19 survey [20]. These data reinforce indications that, although males are at higher risk of developing a severe infection than females [34], the latter are more concerned about COVID-19. This could reflect a stronger adherence to virtuous behaviours in females compared to males [35].

Considering the COVID-19-related variables, during the second survey, fever, headache, myalgia, and olfactory and taste disorders were the most frequent self-reported symptoms among those who tested positive, which are consistently reported as peculiar symptoms associated with SARS-CoV-2 infection [13,14,36]. More than half of the sample underwent the NPS test (positive rate of 13.2%), because of suspected symptoms or contact with a COVID-19 case. More than 40% referred to having shared the workplace in the two weeks before having been tested. About 40% of the sample performed an ST, mostly voluntarily, and 11.9% resulted positive. Taken together, these percentages are significantly higher compared to the official number of positive cases officially reported in Italy for the period March 2020—February 2021 (*n* = 2,925,265 cases in 59,641,488 residents) [2,27], confirming the potential large underestimation of the actual number of exposed or infected. Ideally, only by combining large seroprevalence epidemiological studies (screening tool) with massive NPS testing (diagnostic tool) this issue could be addressed. Most recent Italian serosurveillances still report a very broad range of prevalence estimates. Vena et al. [37] reported 11% IgG and/or IgM positivity in a large adult Italian population between March and April 2020. Among the volunteers recruited in the Marche region from March to June 2020, the authors found a seroprevalence of 14.4%, without significant differences between sex and age groups [38]. As of June 2020, in a population-based study [39] carried out in a northern municipality that was heavily affected by SARS-CoV-2 infection, authors found an overall positivity to SARS-CoV-2 of 22.6%, varying according to age groups. On the other side, the Italian National Institute of Statistics (ISTAT) estimated a much lower seroprevalence of 2.5% in a large sample from 2000 Italian municipalities during the summer of 2020 [40]. Looking at other countries, a systematic review and meta-analysis that included 47 studies involving 399,265 people from 23 countries up to 14 August 2020, reported a seroprevalence that varied from 0.37% to 22.1% in the general population. Limiting the analysis to the Italian dataset, the authors reported a pooled seroprevalence of 7.27 (95%CI 2.48–11.9) and an estimated number of people infected by SARS-CoV-2 of 4,395,587 (95%CI 1,499,457–7,249,393) [41].

Our large-scale data showed no sex difference in the proportion of respondents infected with SARS-CoV-2, in accordance with current knowledge. Although epidemiological evidence in the early phase of pandemic suggested that males had higher risk of SARS-CoV-2 infection than females [42], subsequent evidence demonstrated that this risk difference was not significant [43]. This indicates that unequal access to healthcare and testing between sexes could have skewed towards a male bias in diagnosing the infection during the first wave of the pandemic. On the other hand, males were more frequently hospitalized and possibly manifested a more severe disease than females in the present sample. This is consistent with the large body of literature reporting that males face higher rates of hospitalization, intensive therapy unit admission, and death compared to females [34]. We also observed specific sex-differences in relation to the self-reported COVID-19-like symptoms, in which females tend to systematically over-report symptoms. Because no sex difference in the rate of positivity to the diagnostic or screening test has been observed, a possible explanation might reside in the fact that females were more worried about the health situation and tended to be more prone to the phenomenon of the ‘nocebo effects’ compared to males, as shown in other studies [44,45,46]. In line with available evidence, considering only participants with positive results from NPS and/or ST, males more often reported symptoms, such as fever and cough, known as predictors of worse outcomes [47], whereas females reported more frequently symptoms susceptible to subjective perception (headache, anosmia, dysgeusia, sore throat) and generally associated with less severe infections [48,49].

Females also completed more frequently an anti-COVID-19 vaccination cycle compared to their male counterparts. The sex unbalance can be explained by the fact that among the healthcare workers (representing 80% of those who received both vaccine doses in our sample), 73% were females (data in line with the other European Member States [50]). These results reflect the effects of the Guidelines on the Strategic Plan for COVID-19 Vaccines released on 2 December 2020, by the Ministry of Health. These Guidelines, in fact, recommended starting the vaccination campaign by first selecting specific categories, such as healthcare social workers, residents and staff working in nursing homes, at high risk of infection or of spreading the virus [51]. The low percentage of vaccinated (5.7%) in our sample was expected as the anti-COVID-19 vaccination campaign started at the beginning of January 2021 in Italy, when the survey presented in this manuscript was carried out (15 January–28 February).

Remarkably, out of the 2073 participants with at least one positive ST during the period March 2020–February 2021, 72.8% underwent one or more NPS always with negative results or never performed it. Among those with COVID-19, more than one-third became aware that they contracted the infection without being tested (or having a negative result at the NPS), meaning that a considerable number of undiagnosed cases escaped the detection from surveillance systems and was not officially certified as positive. COVID-19 has caught most countries unprepared and has highlighted the unreadiness of health systems [52]. In particular, during the first wave of the pandemic, most countries encountered difficulties in carrying out diagnostic tests, thus limiting the effectiveness of testing, tracking, and contact tracing [5]. Consequently, the number of SARS-CoV-2 infections in the asymptomatic or subclinical infected individuals was largely undetected [53], thus leading to a considerable underestimation of the number of actual cases [54,55].

This large fraction of people, who had not undergone self-isolation or quarantine, is likely to have contributed to the transmission of SARS-CoV-2 infection and to the spread of the disease outbreaks in small communities such as households [56] during the most severe restrictions period, or such as workplaces [57] when the restrictions were less stringent.

The whole body of the data collected and results described in this manuscript will allow us to investigate a number of interesting topics. Among these, we will focus on the association of behavioural factors or individual characteristics with the infection in the two phases of the study; on the impact of pandemics on stress, sleep, and other lifestyles; and on the hesitancy towards an anti-COVID19 vaccination. We plan to address each of these topics with specific methodologies, which are beyond the scope of this descriptive manuscript, in future publications.

### Limitations and Strengths

This present study has some weaknesses, primarily because the online system and voluntary participation suffers from inherent selection bias and generalizability. Similar to other web-based surveys [8,58,59], some of the characteristics of the sample were not adequately representative of the Italian adult population. Indeed, females, younger, healthier, and wealthier people were more represented in the enrolled sample with respect to the general population. Further, data were self-reported, which might have introduced measurement and recall bias (e.g., survey question misunderstanding, etc.). In addition, the longitudinal design may have led to bias due to the loss of participants during the follow-up period. The response rate to the second survey was 40%, with some differences between included and excluded participants, in particular regarding age, education, employment status, and geographical area of residence.

The present study also has several strengths, including its community-based longitudinal design with two time-point’s measures overlapping with the first and second wave of the epidemic in Italy, thus providing reliable details on the temporal evolution of the symptoms and testing. In addition, although these data were self-reported, almost 50% of the studied sample underwent an NPS or ST, providing an overarching picture of the positivity rate at the population level in a country in which the ability to track COVID-19 cases in real time was limited. The exhaustive data collection on socio-demographic, medical, behavioural, and psychological factors, as well as the large sample size, is a further strength of this study. Lastly, the EPICOVID19 web survey has reached a large sample of adults covering all Italian regions, although the response rate was unbalanced in favour of the northern regions, being the Italian geographical area more dramatically affected by the first wave of the pandemic at the time of enrolment.

## 5. Conclusions

EPICOVID19 is the largest web-based survey released when the first two waves of COVID-19 outbreak occurred in Italy. It offers a unique opportunity to estimate the number of individuals asymptomatic or mild symptomatic at the community level, to explore the factors associated with the SARS-CoV-2 infection, and to evaluate the consequences on health and wellbeing of the COVID-19 pandemic in Italy. The descriptive results of the phase II of the EPICOVID19 survey indicate that the positivity rate among Italian adults in February 2021 varied from 11.9% (ST) to 13.2% (NPS). Furthermore, the study highlights that a relevant fraction of positive cases remained uncertified from the official statistics, which possibly may have contributed to the spread of the virus in the community.

Complementary to the activities of testing and contact tracing, the adoption of participatory online surveys for collecting epidemiological data on a multidimensional scale should be considered strategic to support decision makers in planning evidence-based public health actions to control the spread of SARS-CoV-2 infection.

## Figures and Tables

**Table 1:** Individual characteristics of the study participants by sex (*n* = 41,473).

		Sex at Birth		
	Females *n* = 25,146 (60.6)	Males *n* = 16,327 (39.4)	*p*-Value	Total *n* = 41,473 (100)
	*n* (%)	*n* (%)		*n* (%)
Age (mean ± SD)	49.8 ± 13.0	52.2 ± 14.3	0.000	50.7 ± 13.5
Class of age				
19–29	1700 (6.8)	1027 (6.3)	0.000	2727 (6.6)
30–39	4434 (17.6)	2620 (16.0)		7054 (17.0)
40–49	5815 (23.1)	3209 (19.7)		9024 (21.8)
50–59	6802 (27.1)	3905 (23.9)		10,707 (25.8)
60–69	5017 (20.0)	3628 (22.2)		8645 (20.8)
70–79	1262 (5.0)	1707 (10.5)		2969 (7.2)
80+	116 (0.5)	231 (1.4)		347 (0.8)
Educational level ^a^			0.000	
Low	723 (2.9)	658 (4.0)		1381 (3.3)
Middle	7286 (29.0)	5648 (34.6)		12,934 (31.2)
High	17,137 (68.2)	10,021 (61.4)		27,158 (65.5)
Employment status				
Employed, stable position	15,676 (62.3)	10,448 (64.0)	0.000	26,124 (63.0)
Employed, occasional worker	1056 (4.2)	407 (2.5)		1463 (3.5)
Temporary layoff	379 (1.5)	114 (0.7)		493 (1.2)
Unemployed, as before Jun 2020	1243 (4.9)	271 (1.7)		1514 (3.7)
Unemployed, I lost my employment since Jun 2020	446 (1.8)	193 (1.2)		639 (1.5)
Student	850 (3.4)	583 (3.6)		1433 (3.5)
Retired	3471 (13.8)	3420 (20.9)		6891 (16.6)
Other	2025 (8.1)	891 (5.5)		2916 (7.0)
Working at			0.000	
Workplace	7798 (46.6)	4479 (41.3)		12,277 (44.5)
Home and workplace	6188 (37.0)	4270 (39.3)		10,458 (37.9)
Home	2746 (16.4)	2106 (19.4)		4852 (17.6)
Work category at risk for the infection			0.000	
No	10,242 (61.2)	8331 (76.7)		18,573 (67.3)
Personnel who work indoors with high turnout	896 (5.4)	477 (4.4)		1373 (5.0)
School staff	2880 (17.2)	773 (7.1)		3653 (13.2)
Healthcare workers	2572 (15.4)	951 (8.8)		3523 (12.8)
Other (armed forces, haidressers, pilots, etc)	142 (0.8)	323 (3.0)		465 (1.7)
Deprivation Score ^b^			0.000	
Zero	15,005 (59.7)	10,368 (63.5)		25,373 (61.2)
One	8248 (32.8)	4995 (30.6)		13,243 (31.9)
Two	1704 (6.8)	893 (5.5)		2597 (6.3)
Three	182 (0.7)	68 (0.4)		250 (0.6)
Four	7 (0.0)	3 (0.0)		10 (0.0)
Body Mass Index	23.6 ± 4.0	25.6 ± 3.5	0.000	24.4 ± 3.9
N° of morbidities			0.000	
None	15,369 (61.1)	10,660 (65.3)		26,029 (62.8)
One	6042 (24.0)	3553 (21.8)		9595 (23.1)
Two	2416 (9.6)	1389 (8.5)		3805 (9.2)
Three or more	1319 (5.2)	725 (4.4)		2044 (4.9)
Smoking habit			0.000	
No	14,939 (59.4)	8979 (55.0)		23,918 (57.7)
Former smoker	5538 (22.0)	4545 (27.8)		10,083 (24.3)
Current smoker	4669 (18.6)	2803 (17.2)		7472 (18.0)
Frequency of alcohol beverages between meals			0.000	
Never	5739 (22.8)	2043 (12.5)		7782 (18.8)
<5 times a month	11,409 (45.4)	6763 (41.4)		18,172 (43.8)
2–3 times a week	4240 (16.9)	3337 (20.4)		7577 (18.3)
4–5 times a week	2033 (8.1)	1794 (11.0)		3827 (9.2)
6+ times a week	1725 (6.9)	2390 (14.6)		4115 (9.9)
Self-perceived health status				
Bad or very bad	418 1.7)	221 (1.4)	0.000	639 (1.5)
Adequate	5263 (20.9)	2941 (18.0)		8204 (19.8)
Good or very good	19,465 (77.4)	13,165 (80.6)		32,630 (78.7)
Sleep problems ^c^	2509 (10.0)	850 (5.2)	0.000	3359 (8.1)
Perceived stress ^d^			0.000	
Low	10,748 (44.1)	9550 (61.5)		20,298 (50.9)
Moderate	12,445 (51.1)	5633 (36.3)		18,078 (45.3)
High	1168 (4.8)	343 (2.2)		1511 (3.8)
Fear about COVID-19 pandemic (mean ± SD) ^e^	8.6 ± 3.6	7.9 ± 3.5	0.000	8.4 ± 3.6
Feeling to be sufficiently informed about COVID-19	23,443 (93.2)	15,200 (93.1)	0.607	38,643 (93.2)

^a^ Low: illiterate or primary school; middle: middle or high school; high: university or postgraduate degree. ^b^ Townsend Deprivation Score: 0 (no deprivation) to 4 (high deprivation). ^c^ Jenkins sleep scale >12: high sleep problems. ^d^ Perceived Stress Scale: 0–13: low stress; 14–26: moderate stress; 27–40 high stress. ^e^ Fear score ranges from 0 (low) to 16 (high).

**Table 2:** COVID-19-related variables by sex (*n* = 41,473).

		Sex at Birth		
	Females *n* = 25,146 (60.6)	Males *n* = 16,327 (39.4)	*p*-Value	Total *n* = 41,473 (100)
	*n* (%)	*n* (%)		*n* (%)
Close contact with COVID-19 cases			0.000	
No	17,356 (69.0)	11,944 (73.2)		29,300 (70.6)
Yes, wearing a face mask	4630 (18.4)	2504 (15.3)		7134 (17.2)
Yes, at least once without wearing a face mask	3160 (12.6)	1879 (11.5)		5039 (12.2)
Quarantine or self-isolation			0.000	
Never	17,441 (69.4)	11,834 (72.5)		29,275 (70.6)
Once	6500 (25.8)	3768 (23.1)		10,268 (24.8)
More than once	1205 (4.8)	725 (4.4)		1930 (4.7)
NPS test for SARS-CoV-2 ^ *			0.000	
Not done	11,592 (46.1)	8004 (49.0)		19,596 (47.3)
Yes, always negative	11,792 (46.9)	7183 (44.0)		18,975 (45.8)
Yes, positive at least once	1762 (7.0)	1140 (7.0)		2902 (7.0)
If tested, number of NPS			0.071	
1	5715 (42.2)	3615 (43.4)		9330 (42.6)
2	3129 (23.1)	1948 (23.4)		5077 (23.2)
3	1902 (14.0)	1148 (13.8)		3050 (13.9)
4+	2808 (20.7)	1612 (19.4)		4420 (20.2)
Molecular NPS test type	1590 (90.2)	995 (87.3)	0.030	2585 (89.1)
NPS test performed for free	1405 (79.7)	870 (76.3)	0.029	2275 (78.4)
Reasons for the positive NPS test performed				
Presence of symptoms	1124 (63.8)	748 (65.6)	0.316	1872 (64.5)
Contact with COVID-19 case	827 (46.9)	455 (39.9)	0.000	1282 (44.2)
Check at workplace	205 (11.6)	93 (8.2)	0.003	298 (10.3)
Own choice	79 (4.5)	77 (6.8)	0.008	156 (5.4)
Other reasons	86 (4.9)	69 (6.1)	0.170	155 (5.3)
Places attended two weeks before the positive NPS test				
School	215 (12.2)	54 (4.7)	0.000	269 (9.3)
Bar/restaurants	408 (23.2)	373 (32.7)	0.000	781 (26.9)
Gym/swimming pool/club/discotheques	125 (7.1)	84 (7.4)	0.780	209 (7.2)
Churches	175 (9.9)	98 (8.6)	0.229	273 (9.4)
Hairdresser/aesthetic centre	223 (12.7)	45 (3.9)	0.000	268 (9.2)
Theatres/cinemas/museum	35 (2.0)	23 (2.0)	0.953	58 (2.0)
Parties (friends, family)	298 (16.9)	241 (21.1)	0.004	539 (18.6)
Public transports>3 times/week	132 (7.5)	82 (7.2)	0.764	214 (7.4)
Shared workplace	759 (43.1)	440 (38.6)	0.017	1199 (41.3)
Hospitalization after NPS positive test	180 (10.2)	179 (15.7)	0.000	359 (12.4)
ST for SARS-CoV-2 **			0.000	
Not done	14,054 (55.9)	10,025 (61.4)		24,079 (58.1)
Yes, always negative	9788 (38.9)	5533 (33.9)		15,321 (36.9)
Yes, positive at least once	1304 (5.2)	769 (4.7)		2073 (5.0)
Reasons for the positive ST performed				
Check at workplace	450 (34.5)	188 (24.4)	0.000	638 (30.8)
Own choice	594 (45.6)	440 (57.2)	0.000	1034 (49.9)
Other reasons	311 (23.8)	168 (21.8)	0.296	479 (23.1)
Vaccinated for COVID-19 at 2nd interview			0.000	
No	21,967 (87.4)	14,853 (91.0)		36,820 (88.8)
Yes, only the first dose	1540 (6.1)	742 (4.5)		2282 (5.5)
Yes, both doses	1639 (6.5)	732 (4.5)		2371 (5.7)

* NPS: nasopharyngeal swab; ^ SARS-CoV-2: severe acute respiratory syndrome coronavirus 2; ** ST: serological test.

## Data Availability

Data are available on request.

## Supplementary Materials

The following supporting information can be downloaded at: https://www.mdpi.com/article/10.3390/ijerph19031274/s1, Annex 1: Phase II EPICOVID19 questionnaire; Table S1: Contents of the web-based questionnaires; Table S2: Validated scales and questionnaires used in EPICOVID19 survey; Table S3: Characteristics of individuals who participated in phase II survey and of those who did not; Table S4: Standardized response rates per 100,000 inhabitants by Italian region over the phase II study period; Table S5: Frequency of self-reported symptoms by sex and by positivity to NPS and/or ST between March 2020 and February 2021. Membership of the EPICOVID19 Working Group.
