# Supplementary material for: Epidemiology of SARS-CoV-2 Infection in Italy Using Real-World Data: Methodology and Cohort Description of the Second Phase of Web-Based EPICOVID19 Study
Source: Int J Environ Res Public Health. 2022 Jan 24;19(3):1274. doi: 10.3390/ijerph19031274 (PMC8835202; doi:10.3390/ijerph19031274)
Supplement: Supplementary file 1 [file ijerph-19-01274-s001.zip › ANNEX 1 - Phase II EPICOVID19 questionnaire.pdf]

QUESTIONNAIRE EPICOVID19 – PHASE 2

SOCIO-DEMOGRAPHIC DATA

**Mandatory questions*

1. **Sex at birth*** M |__| F |__| Prefers not to answer |__|

2. **Province of domicile*** |__|__|__|__|__|__|__|__|__|__|__|__|__|__|__|__|__|__|__|__|__|__|__|

3. **Municipality*** |__|__|__|__|__|__|__|__|__|__|__|__|__|__|__|__|__|__|__|__|__|__|__|__|__|

4. **Postal code*** |__|__|__|__|__|

5. **Indicate your employment status from the beginning of June 2020 to the beginning of the restrictive measures in November:***
|__| Employed, steady employment
|__| Employed, occasional worker (e.g. on-call work)
|__| Student
|__| Unemployed now as well as before the reference period
|__| Unemployed now because I lost my job in the meantime
|__| Retired
|__| Temporary layoff
|__| None of the above

6. *If the answer is yes to one of the first two answers:**
|__| I resumed working exclusively on-site, at my workplace
|__| I work from home only
|__| I alternate work from home with on-site work

7. **In the last 6 months (from the beginning of June 2020 to date), did your occupation include, even partially, one of the following? (multiple choices are possible)***
|__| Healthcare workers in close contact with the patient (e.g. clinician, nurses, dentists, social health operator, rescuers)
|__| School staff who works in the presence of children or teenagers (teachers, educators, cleaning staff)
|__| Personnel who work in highly crowded rooms or closed environments (e.g. supermarkets, bars, restaurants, gyms, swimming pools, shops, clubs, discos)
|__| Armed forces occupations (e.g. policeman, soldier)
|__| Bus and tram drivers, flight attendants, taxi drivers
|__| Staff working in beauty salons and hairdressers
|__| None of the above

CLINICAL FEATURES AND COVID-19 RELATED VARIABLES

8. **Weight*** |__|__|__| Kg

9. **Height*** |__|__|__| cm

10. **Please indicate your blood type***
|__| A
|__| B
|__| AB
|__| O
|__| I don't know

11. Do you have or have you had one or more of the following symptoms? If yes, indicate when (more choices are possible)*

**Symptoms**	**Yes**	**Before September 2020**	**September 2020**	**October 2020**	**November 2020**	**December 2020**	**January 2021**
Fever with a temperature greater than 37.5 °C for at least one day							
Cough							
Sore throat/rhinorrhoea							
Headache							
Myalgia							
Taste disorder							
Olfactory disorder							
Difficulty breathing, shortness of breath due to light exertion (e.g. walking or climbing a few steps) and / or shortness of breath at rest							
Chest pain (sternum pain)							
Gastrointestinal disorders (diarrhoea, nausea, vomiting)							
Dermatological manifestations (rashes)							
Loss of appetite							
Heart arrhythmia, palpitations, tachycardia, chest tightness							
Other neurological disorders (dizziness, confusion, memory problems)							
No symptoms							

12. If you have had symptoms attributable to a suspected or ascertained infection (swab and/or serological test), please indicate for each one whether:

**Symptoms**	**I have fully recovered**	**I partially recovered**	**I have not recovered at all**
Fever with a temperature greater than 37.5 °C for at least one day			
Cough			
Sore throat/rhinorrhoea			
Headache			
Myalgia			
Taste disorder			
Olfactory disorder			
Difficulty breathing, shortness of breath due to light exertion (e.g. walking or climbing a few steps) and / or shortness of breath at rest			
Chest pain (sternum pain)			
Gastrointestinal disorders (diarrhoea, nausea, vomiting)			
Dermatological manifestations (rashes)			
Loss of appetite			
Heart arrhythmia, palpitations, tachycardia, chest tightness			
Other neurological disorders (dizziness, confusion, memory problems)			

13. Please indicate if you have or have been diagnosed with one or more of these diseases or disorders*

**Diseases or ailments**	**Before June 2020**	**From June 2020 to date**
Lung diseases (e.g. asthma, obstructive pulmonary disease, metapneumonic fibrosis)		
Heart diseases (e.g. ischemic heart disease, atrial fibrillation, heart failure)		
Hypertension		
Kidney diseases or dialysis		
Immune system diseases (e.g. celiac disease, Graves' disease, thyroid disease, psoriasis, rheumatoid arthritis, systemic lupus erythematosus, type I diabetes)		
Tumours		
Metabolic diseases (e.g. type II diabetes, obesity, gout)		
Neurological diseases(e.g. multiple sclerosis, epilepsy, neuropathies)		
Cerebrovascular diseases (e.g. stroke, ischemia)		
Hepatitis B		
Hepatitis C		

Other liver diseases (e.g. liver cirrhosis, liver failure)		
Depression and/or anxiety		
Eating disorders (bulimia, anorexia, or other eating disorders)		
Anaemia		
No disease or disorder		

14. Please indicate if these conditions have occurred during your life*

Other conditions	
Not self-sufficient in carrying out daily activities (e.g. washing, dressing, shopping, cooking)	
Allergies to pollens	
Food allergy	
Recurrent herpes	
Cytomegalovirus infection	
Papilloma virus infection	
None of these conditions	

15. Indicate if you regularly use these medications or supplements (not as a treatment for COVID-19):*

|__| Oral or injection cortisones
|__| Inhaled cortisones and/or bronchodilators
|__| Antidepressants/anxiolytics/sedatives
|__| Vitamin D (e.g. DIBASE)
|__| Multivitamins

16. Did you have the following vaccinations?*

Vaccinations	
Anti-flu in the winter season 2020/2021	
Anti-pneumococcal in the past 5 years	
Anti-zoster	
Anti-meningococcal	
Anti-tetanus in the past 10 years	
None of these vaccinations	

17. Did you get the COVID19 vaccination?*

|__| No
|__| Yes, only the first dose
|__| Yes, both doses

To be completed only if answer 17 is "Yes, only the first dose" or "Yes, both doses"

18. Indicate the date of the first vaccine dose?*

|__|__| /|__||__|/|__||__||__||__|

To be completed only if answer 17 is “No”

19. Thinking about the COVID-19 vaccine:

|__| I will definitely get the vaccine
|__| I will probably get the vaccine, but before that I will inform myself better

|__| I will probably not get the vaccine, but in any case I will inform myself better
|__| I will not get the vaccine
|__| I can't answer at this moment

****

To be completed only if of female sex

20. Are you pregnant?*
|__| No |__| Yes |__| I'm in menopause

21. Indicate the number of completed pregnancies*
|__| 0 |__| 1 |__| 2 or more

22. Have you started taking contraceptives/birth pills from 1 June 2020 to date?*
|__| No
|__| Yes

23. Have you started taking hormone replacement therapy from 1 June 2020 to date?*
|__| No
|__| Yes

To be completed only if of male sex

24. Are you following or have you been following hormone therapy (e.g. anti-androgen)?*
|__| Never
|__| Yes, in the past, for less than 5 years
|__| Yes, in the past, for more than 5 years
|__| Yes currently, taking it less than 5 years
|__| Yes currently, taking it more than 5 years

****

25. Have you been in a close contact (direct contact at a distance of less than 2 meters, and lasting more than 15 minutes in a closed environment such as a house, workplace, waiting room, airplane, transportation vehicles) with confirmed COVID-19 cases, live or deceased?*
|__| No, as far as I know, I have not been in a close contact
|__| Yes, at least once without the mask
|__| Yes, at least once but always with a mask

26. Have you ever been in fiduciary isolation (or quarantine) either self-imposed or established by the health authorities?*
|__| Never
|__| Yes, once
|__| Yes, more than once

<u>NASOPHARYNGEAL SWAB FOR COVID-19</u>

27. Since September 1, have you had at least one swab for COVID-19?*
|__| No, I did not perform any test
|__| Yes, at least once with a positive result

|__| Yes, always with a negative result

If the answer is "Yes" to the last two answers:

28. How many swab tests did you take?*
|__| 1
|__| 2
|__| 3
|__| 4 or +

To be completed only if answer 27 is "Yes, at least once with a positive result"

Considering the first swab with a positive result, indicate:

29. Month in which it was performed* |__|__|__|__|__|__|

30. Type of swab test*
|__| Molecular/PCR (outcome after at least 24 hours)
|__| Rapid (results in less than one hour)
|__| I do not know

31. Did you pay for the swab?*
|__| No |__| Yes

32. Reason for swabbing (multiple choices are possible)*
|__| I had developed suspicious symptoms
|__| I have had close contact with a confirmed COVID-19 case (in the home, workplace, etc.)
|__| For workplace control (e.g. healthcare workers)
|__| Personal choice
|__| Other reasons than those listed above

To be completed only if you have had symptoms

33. Number of days elapsed between the onset of symptoms and the swab test*
|__| 1-2 days
|__| 3-7 days
|__| 8-14 days
|__| More than 14 days

34. In the two weeks before the swab, did you attend one or more of these places? (multiple choices are possible)*
|__| Schools
|__| Bars, restaurants
|__| Gyms, swimming pools, clubs, discotheques
|__| Churches, oratories
|__| Beauty center, hairdresser
|__| Theatres, cinemas, museums
|__| Parties, lunches, dinners at home
|__| Public transport more than 3 times a week
|__| Shared working spaces in less than two meters away with at least one other person

35. Did you ever go to the hospital after the infection?*
|__| Never
|__| Yes, but I was sent back and treated at home
|__| Yes, I was hospitalized and underwent pharmacological treatments with or without oxygen in the ward with a simple mask or nasal glasses but without the need for a helmet / CPAP

|__| Yes, I was hospitalized and underwent pharmacological treatment with or without oxygen with non-invasive ventilation (e.g. helmet / CPAP)
|__| Yes, I was hospitalized and underwent to pharmacological treatments and intubation

To be completed only in case of hospitalization

36. How many days did the hospitalization last?* |__||__||__| days

37. What treatments did you undergo for the treatment of COVID-19? (multiple choices are possible)*
|__| Low molecular weight heparin
|__| Cortisones
|__| Azithromycin
|__| Other antibiotics
|__| Antipyretics or pain relievers
|__| Remdesevir
|__| Lopinavir / ritonavir (Kaletra)
|__| Clorochina
|__| Oxygen therapy
|__| Other or do not know
|__| None

38. Did you have at least one control swab test?*
|__| Yes, and my last swab is positive ==> Please indicate the number of days between the first positive swab and the control test | ___ || ___ |
|__| Yes, and my last swab is negative ==> Please indicate the number of days between the first positive swab and the control test | ___ || ___ |

|__| No, I haven't done it ==> Has it been more than a month since the first positive swab to date?

| __ | No | __ | Yes

To be completed only if answer 24 is "Yes, always with a negative result"

If you have always swabs with negative results, think about your last negative swab and indicate:

39. Month in which it was made* |__|__|__|__|__|

40. Type of swab test*
|__| Molecular /PCR (outcome after at least 24 hours)
|__| Rapid (results in less than one hours)
|__| I do not know

41. Did you have to pay for the swab?*
|__| No |__| Yes

42. Reason for swabbing (multiple choices are possible)*
|__| I had developed suspicious symptoms
|__| I have had close contact with a confirmed COVID-19 case (at home, workplace, etc.)
|__| For workplace control (e.g. health care workers)
|__| Personal choice
|__| Other reasons than those listed above

TO BE COMPLETED ONLY IF HAS SYMPTOMS

43. Number of days elapsed between the onset of symptoms and the swab test*
|__| 1-2 days
|__| 3-7 days
|__| 8-14 days

|__| More than 14 days

44. In the two weeks before the swab, did you attend one or more of these places? (multiple choices are possible)*
|__| Schools
|__| Bars, restaurants
|__| Gyms, swimming pools, clubs, discotheques
|__| Churches, oratories
|__| Beauty center, hairdresser
|__| Theatres, cinemas, museums
|__| Parties, lunches, dinners at home
|__| Public transport more than 3 times a week
|__| Shared working spaces in less than two meters away with at least one other person

<u>SEROLOGICAL TEST FOR COVID-19</u>

45. Since September 1st, have you done at least one serological test (blood drop or blood sample) for COVID-19?*
|__| No, I did not perform any test
|__| Yes, at least once with a positive result
|__| Yes, always with a negative result

To be completed only if the answer is "Yes, at least once with a positive result"

46. Thinking about the first (or the only) positive serological test, indicate the month in which it was performed* |__||__||__||__||__||__|

47. Type of test* |__| Lancing device |__| Blood sample

48. Reason for doing the serological test (multiple choices are possible)*
|__| For workplace control (e.g. healthcare workers)
|__| Personal choice
|__| Other reasons than those listed above

49. After the first positive serological test, have you ever had a negative serological test?*
|__| No, I've never done any other serological tests
|__| No, my last serological test is still positive => Last month positive test | ___ || ___ || ___ || ___ |
|__| Yes => Month of first negative test | ___ || ___ || ___ || ___ |

To be completed only if the answer is "Yes, always with a negative result"

50. Thinking about the last negative serological test, indicate the month in which it was performed* |__||__||__||__||__||__|

51. Type of test* |__| Lancing device |__| Blood sample

52. Reason for doing the serological test (multiple choices are possible)*
|__| For workplace control (e.g. healthcare workers)
|__| Personal choice
|__| Other reasons than those listed above

QUESTIONNAIRE EPICOVID19 – PHASE 2

PERSONAL CHARACTERISTICS AND HEALTH STATUS

53. How would you describe your health in general?*
|__| Very bad |__| Bad |__| Adequate |__| Good |__| Very good

54. After the COVID-19 pandemic situation:*

	No	A little bit	Neutral	Enough	Yes
Are you afraid of being infected with COVID-19?					
Are you afraid that your family members might be infected?					
Are you worried about your economic and working conditions?					
Are you worried about the economic and working conditions of your family members?					

55. How many days in the past 30 days did you:*

	0	1	2	3	4	5
Have trouble falling asleep?						
Wake up several times per night but did not have trouble falling asleep again?						
Wake up one or more times per night (including waking far too early) and have trouble falling asleep again?						
Wake up after your usual amount of sleep feeling tired or exhausted?						

0 = Not at all 1 = 1-3 days 2 = 4-7 days 3 = 8-14 days 4 = 15-21 days 5 = 22-30 days

56. Thinking about the last month, how often:*

	0	1	2	3	4
Have you been upset because of something that happened unexpectedly?					
Have you felt that you were unable to control the important things in your life?					
Have you felt nervous and “stressed”?					
Have you felt confident about your ability to handle your personal problems?					
Have you felt that things were going your way?					
Have you found that you could not cope with all the things that you had to do?					
Have you been able to control irritations in your life?					
Have you felt that you were on top of things?					
Have you been angered because of things that were outside of your control?					
Have you felt difficulties were piling up so high that you could not overcome them?					
Have you trusted yourself?					
Have you trusted others?					
Have you felt you can protect your loved ones?					
Have you felt you can help improve the situation?					
Have you felt helpless?					

0 = Never 1 = Almost never 2 = Sometimes 3 = Fairly often 4 = Very often

LIFESTYLES AND BEHAVIORS

57. Do you smoke cigarettes (including electronic cigarettes)?*
|__| I don't smoke, or have smoked less than 100 cigarettes in my lifetime, and I don't currently smoke
|__| I am a former-smoker (I have smoked at least 100 cigarettes in my lifetime, but I do not smoke anymore)
|__| Yes, smoking less than 10 cigarettes per day
|__| Yes, smoking between 10 and 20 cigarettes per day
|__| Yes, smoking more than 20 cigarettes a day (more than one pack a day)

To be completed only if former smoker

58. At what age did you quit smoking?* __________ years
59. At what age did you start smoking?* ___________ years
60. How many cigarettes a day did you smoke before quitting?*
|__| I smoked less than 10 cigarettes a day
|__| I smoked between 10 and 20 cigarettes a day
|__| I smoked more than 20 cigarettes a day (more than one pack a day)

61. Why did you quit smoking?*
|__| Health problems
|__| Doctor's advice
|__| Personal choice
|__| Illness of relatives or friends

To be completed only if current smoker

62. At what age did you start smoking?* __________ years

63. Have you ever smoked or are you currently smoking electronic cigarettes?*
|__| No
|__| Yes, exclusively electronic cigarettes
|__| Yes, not exclusively electronic cigarettes (including tobacco cigarettes)

64. Does someone usually smoke in your presence in closed environments (at home, in the car, etc.)*
|__| No
|__| Yes, one person
|__| Yes, more than one person

65. Thinking about the last year, if you have consumed alcohol with meals or between meals, please indicate the frequency*
|__| I don't drink alcohol
|__| Once a month
|__| 2-4 times a month
|__| 2-3 times a week
|__| 4-5 times a week
|__| 6 or more times a week

66. Since the pandemic started (from February 2020), please indicate if (and how) the following habits or conditions have changed*

	Not present or not applicable	Decreased	Unchanged	Increased
Snacking habits between meals				

Skip one of the main meals				
Daily consumption of fruit and vegetables				
Eating a balanced diet (including healthy ingredients such as whole grains, legumes, eggs, nuts, fruits and vegetables)				
Consumption of sugary foods, drinks, ice cream, sweets, candies				
Consumption of fried food/fast food				
Daily/weekly alcohol consumption (during and/or away from meals)				
Physical activity involving an increase in heart rate and breathing (brisk walking, cycling, swimming, running)				
Time spent sitting and in front of the screen (TV, computer, mobile phone)				
Hours of sleep				
Access to the National Health Service (SSN) for visits and examinations for minor health problems (preventive visits or checks)				
Access to the National Health Service (SSN) for visits and examinations for major health problems (oncologic, major surgeries, withdrawing of chronic life-sustaining treatments, heart failure, BPCO)				
Contact with the general practitioner				

HOUSING CONDITIONS

67. What type of heating do you mainly use in your home?*
|__| None
|__| Radiator
|__| Panels (floor, walls)
|__| Fan-coil/split/heat pump/fan coil units
|__| Gas stove
|__| Electric stove
|__| Kerosene stove
|__| Pellet stove
|__| Fireplace/stove or wood boiler

68. How many rooms are there in your home (excluding the bathroom and auxiliary spaces)?*
|__| One |__| Two |__| Three |__| More than three

69. Besides you, how many people live in your household?*
|__| None |__| One |__| Two |__| More than two

70. Are there minors (<18 years old) among your cohabitants?*
|__| No
|__| Yes, one
|__| Yes, two
|__| Yes, three or more

71. The house you currently live in is *
|__| Owned |__| Rented |__| Owned by relatives |__| Other (social housing, cooperative housing)

72. How many cars does your household own?*
0 |__| 1|__| 2 |__| 3 or more |__|

SOURCES OF INFORMATION ON COVID-19

73. Do you feel sufficiently informed about the risks of COVID-19?
|__| No |__| Yes

74. What sources of information have you consulted / listened about the risks of COVID-19 and how reliable do you consider them?

Information source	No	Yes, and I don't trust it	Yes, and I partially trust it	Yes, and I trust it
Television (national and/or local)				
Newspapers and magazines				
Radio (national and/or local)				
Government, Ministries, Departments, Civil Protection, Law Enforcement, European Union				
Newspaper, TV or radio websites				
Scientists, researchers, academics				
Scientific publications online				
Associations or trade unions (environmentalists, volunteers)				
Religious institutions				
Search engines (Google)				
Social media channels (Facebook, Twitter, Instagram, WhatsApp)				

75. Below are listed some problems you may encounter in everyday life. What do you think is risky? Is it low, medium or high risk?

Problems	Low	Medium	High	I do not know
Natural disasters (earthquakes, floods, etc.)				
Unemployment				
Pollution of the environment				
Epidemics				
Poverty				
Terrorism				
Climate changes				
Crime				
Contaminated food				

Any additional information, reports and comments

____

____

____

QUESTIONNAIRE EPICOVID19 – PHASE 2

EXPRESSION OF INTEREST

Do you agree to be contacted to participate in an Italian study promoted by the National Research Council, which provides for free swabs and the collection of a biological sample (saliva/a few drops of blood)? Among those who join, a random sample will be selected to participate in the national study.

|__| No
|__| Yes

Do you accept to be contacted for future phases of the EPICOVID19 study?

|__| No
|__| Yes

If you agree, please let us know if you want to be contacted at another e-mail address other than the one we already used to contact you back

@____

and / or a telephone number

____

To receive information about the project, you can contact the research team at this email address epicovid19@itb.cnr.it or follow the updates on our website https://epicovid19.itb.cnr.it/
