# Supplementary material for: Epidemiology of SARS-CoV-2 Infection in Italy Using Real-World Data: Methodology and Cohort Description of the Second Phase of Web-Based EPICOVID19 Study
Source: Int J Environ Res Public Health. 2022 Jan 24;19(3):1274. doi: 10.3390/ijerph19031274 (PMC8835202; doi:10.3390/ijerph19031274)
Supplement: Supplementary file 1 [file ijerph-19-01274-s001.zip › Membership EPICOVID19 Working Group.pdf]

Membership of the EPICOVID19 Working Group.

Fulvio Adorni	MPH	fulvio.adorni@itb.cnr.it	National Research Council, Institute of Biomedical Technologies, Via Fratelli Cervi 93, 20054 Segrate (MI), Italy
Massimo Andreoni	MD	andreoni@uniroma2.it	Infectious Diseases Clinic, Department of System Medicine, Tor Vergata University of Rome, 00133 Rome, Italy
Raffaele Antonelli Incalzi	MD	r.antonelli@unicampus.it	Unit of Geriatrics, Department of Medicine, Biomedical Campus of Rome, via Alvaro del Portillo, 21, 00128 Rome, Italy
Luca Bastiani	PhD	luca.bastiani@ifc.cnr.it	National Research Council, Institute of Clinical Physiology, Via G. Moruzzi 1, 56124 Pisa (PI), Italy
Fabrizio Bianchi	PhD	fabriepi@ifc.cnr.it	National Research Council, Institute of Clinical Physiology, Via G. Moruzzi 1, 56124 Pisa (PI), Italy
Di Bari Mauro	MD	mauro.dibari@unifi.it	Geriatric Intensive Care Medicine, University of Florence and Azienda Ospedaliero-Universitaria Careggi, Largo Brambilla, 3 - 50134 Firenze, Italy
Loredana Fortunato	MSc	loredana.fortunato@ifc.cnr.it	National Research Council, Institute of Clinical Physiology, Via G. Moruzzi 1, 56124 Pisa (PI), Italy
Massimo Galli	MD	massimo.galli@unimi.it	Infectious Diseases Unit, Department of Biomedical and Clinical Sciences L.Sacco, Università di Milano, ASST Fatebenefratelli Sacco, Via G.B. Grassi 74, 20157 Milano, Italy
Andrea Giacomelli	MD	andrea.giacomelli@unimi.it	Infectious Diseases Unit, Department of Biomedical and Clinical Sciences L.Sacco, Università di Milano, ASST Fatebenefratelli Sacco, Via G.B. Grassi 74, 20157 Milano, Italy
Nithiya Jesuthasan	MPH	nithiya.jesuthasan@itb.cnr.it	National Research Council, Institute of Biomedical Technologies, Via Fratelli Cervi 93, 20054 Segrate (MI), Italy
Stefania Maggi	MD; PhD	stefania.maggi@in.cnr.it	National Research Council, Institute of Neuroscience, Aging Branch, Via Vincenzo Maria Gallucci 16, 35128 Padova
Claudio Maria Mastroianni	MD	claudio.mastroianni@uniroma1.it	Department of Public Health and Infectious Disease, "Sapienza" University, Piazzale Aldo Moro 5, 00185 Roma, Italy.

Sabrina Molinaro	PhD	sabrina.molinaro@ifc.cnr.it	National Research Council, Institute of Clinical Physiology, Via G. Moruzzi 1, 56124 Pisa (PI), Italy
Marianna Noale	MSc	marianna.noale@in.cnr.it	National Research Council, Institute of Neuroscience, Aging Branch, Via Vincenzo Maria Gallucci 16, 35128 Padova
Gabriele Pagani	MD	gabriele.pagani@asst-ovestmi.it	Infectious Diseases Unit, Legnano General Hospital, ASST Ovest Milanese, 20025 Legnano (MI), Italy and DIBIC "Luigi Sacco", University of Milan, 20157 Milan, Italy
Claudio Pedone	MD	claudio.pedone@gmail.com	Unit of Geriatrics, Department of Medicine, Biomedical Campus of Rome, Via Álvaro del Portillo 21, 00128 Roma, Italy
Carla Pettenati	MD	cpettenati@me.com	National Research Council, Institute of Biomedical Technologies, Via Fratelli Cervi 93, 20054 Segrate (MI), Italy
Federica Prinelli	PhD	federica.prinelli@itb.cnr.it	National Research Council, Institute of Biomedical Technologies, Via Fratelli Cervi 93, 20054 Segrate (MI), Italy
Stefano Rusconi	MD	stefano.rusconi@unimi.it	Infectious Diseases Unit, Legnano General Hospital, ASST Ovest Milanese, 20025 Legnano (MI), Italy and DIBIC "Luigi Sacco", University of Milan, 20157 Milan, Italy
Aleksandra Sojic	PhD	aleksandra.sojic@itb.cnr.it	National Research Council, Institute of Biomedical Technologies, Via Fratelli Cervi 93, 20054 Segrate (MI), Italy
Marcello Tavio	MD	marcello.tavio@ospedaliriuniti.marche.i t	Division of Infectious Diseases, Azienda Ospedaliero Universitaria Ospedali Riuniti, Via Conca, 71 - 60126 Torrette - Ancona, Italy
Caterina Trevisan	MD, PhD	caterina.trevisan.5@studenti.unipd.it	University of Padova, Department of Medicine (DIMED) – Geriatric Unit, Via Giustiniani 2, 35128 Padova (PD), Italy and Department of Medical Sciences, University of Ferrara, via Aldo Moro 8, 44124, Cona - Ferrara, Italy
