# Supplementary material for: Epidemiology of SARS-CoV-2 Infection in Italy Using Real-World Data: Methodology and Cohort Description of the Second Phase of Web-Based EPICOVID19 Study
Source: Int J Environ Res Public Health. 2022 Jan 24;19(3):1274. doi: 10.3390/ijerph19031274 (PMC8835202; doi:10.3390/ijerph19031274)
Supplement: Supplementary file 1 [file ijerph-19-01274-s001.zip › Supplementary material.pdf]

Table S1. Contents of the web-based questionnaires.

Questionnaires' sections	EPICOVID19 phase I	EPICOVID19 phase II
Socio-demographic characteristics		
Date of birth	✓	
Sex	✓	✓
Country and province of residency	✓	✓
Ethnicity	✓	
Educational attainment	✓	
Occupational level	✓	✓
Employment status	✓	✓
Clinical features and COVID-19 related variables		
Weight		✓
Height		✓
Blood type		✓
COVID-19-like symptoms	✓	✓
Presence of chronic diseases or conditions	✓	✓
Use of drugs or supplement	✓	✓
Vaccines	✓	✓
COVID-19 vaccine		✓
Use of birth pills and/or hormone replacement therapy	✓	✓
Currently and past pregnancy	✓	✓
Anti-androgens hormone therapy		✓
Contacts with COVID-19 cases	✓	✓
Fiduciary isolation (or quarantine)		✓
COVID-19 nasopharingeal swab testing	✓	✓
Date of COVID-19 nasopharingeal swab testing		✓
Type of COVID-19 nasopharingeal swab testing		✓
Reasons for COVID-19 nasopharingeal swab testing		✓
Time from symptoms		✓
COVID-19 serological testing		✓
Date of COVID-19 serological testing		✓
Type of COVID-19 serological testing		✓
Reasons for COVID-19 serological testing		✓
Contact with emergency number	✓	✓
Hospitalization for COVID-19	✓	✓
Personal characteristics and behaviours		
Self-reported health status	✓	✓
Fear of SARS-CoV-2^ infection	✓	✓
Sleep disorders		✓
Anxiety and stress perceived		✓
Change of behaviours during the confinement		✓
Population density in the area of residence	✓	
Traffic intensity	✓	
Number of co-habitants	✓	✓
Number of rooms	✓	✓
Home heating system		✓

Presence of at-risk co-habitants	✓	
Presence of co-habitants aged less than 18 years		✓
Daily mean number of contacts	✓	
Smoking habit	✓	✓
Alcoholic beverages		✓
Physical activity	✓	
Job condition	✓	
Going out weekly	✓	
Use of public transportation	✓	
House property		✓
Cars property		✓
Number of cars		✓
COVID-19 sources of information		✓
Risk perceived		✓

^SARS-CoV-2: severe acute respiratory syndrome coronavirus 2.

Table S2. Validated scales and questionnaires used in EPICOVID19 survey

**Validated scales and questionnaires**	**Measures**
Educational attainment	http://uis.unesco.org/sites/default/files/documents/international-standard-classification-of-education-isced-2011-en.pdf
Occupational status	https://www.eurofound.europa.eu/surveys/ewcs/2005/classification
Self-perceived health status	https://ec.europa.eu/eurostat/statistics-explained/index.php?title=Glossary:Self-perceived_health
Risk perception	Kim, J.S.; et al. Middle East respiratory syndrome-related knowledge, preventive behaviours and risk perception among nursing students during outbreak. J Clin Nurs. 2016 Sep;25(17-18):2542-9.
Clinical features	World Health Organization & International Severe Acute Respiratory and emerging Infection Consortium. (2020). Global COVID-19 Clinical Platform Novel Coronavirus (COVID-19)- Rapid Version, "https://www.who.int/docs/default-source/coronaviruse/who-ncov-crf.pdf?sfvrsn=84766e69_2.," [Online].<br>Case Record Form Instructions Severe Acute Respiratory Infection Clinical Characterization Data Tool, " https://media.tghn.org/medialibrary/2019/06/SPRINT-SARI_CRF.V2_Dec_2017__Complete.pdf," [Online].<br>M. E. R. S. C. (MERS-CoV), "https://www.who.int/csr/disease/coronavirus_infections/MERS_case_investigation_questionnaire.pdf?ua=1.," [Online].
Self-reported traffic intensity	Cesaroni, G.; Badaloni, C.; Porta, D.; Forastiere, F.; et al. Comparison between various indices of exposure to traffic-related air pollution and their impact on respiratory health in adults. Occup Environ Med. 2008 Oct;65(10):683-90.
Physical activity	Centers for Disease Control and Prevention, "Strategies to Prevent Obesity and Other Chronic Diseases: The CDC Guide to Strategies to Increase Physical Activity in the Community. Atlanta: U.S. Department of Health and Human Services; 2011.
Sleeping disorders	Jenkins, C.D.; Stanton, B.A.; Niemcryk, S.J.; Rose, R.M. A scale for the estimation of sleep problems in clinical research. J Clin Epidemiol 1988;41(4):313-21
Smoking habit	National Health Interview Survey – Adult tobacco use information. Centers for Disease Prevention and Control. 2017 Aug 29 https://www.cdc.gov/nchs/nhis/tobacco/tobacco_glossary.htm
Perceived stress	Cohen, S.; Kamarck, T.; and Mermelstein, R. A global measure of perceived stress. Journal of Health and Social Behavior. 1983, 24, 386-396 (adapted)
Alcohol consumption	Frank, D.; De Benedetti, A.F.; Volk, R.J.; Williams, E.C.; Kivlahan, D.R.; Bradley, K.A. Effectiveness of the AUDIT-C as a screening test for alcohol misuse in three race/ethnic groups. J Gen Intern Med. 2008, 23(6):781-787 (adapted)
Deprivation index	Townsend, P.; Phillimore, P.; Beattie, A. (1988) Health and Deprivation: Inequality and the North Croom Helm: London

Table S3. Characteristics of individuals who participated in phase II survey (*n*=41,473) and of those who did not (*n*=157,349)

		Phase II survey		
		**Participated (*n*=41,473)**	**Did not participate (*n*=157,349)**	***p*-value**
Sex	Females	25,146 (60.6)	93,524 (59.4)	
	Males	16,327 (39.4)	63,825 (40.6)	<0.001
Class of age	18-29	3,255 (7.8)	21,564 (13.7)	
	30-39	7,336 (17.7)	30,427 (19.3)	
	40-49	9,274 (22.4)	33,468 (21.3)	
	50-59	10,616 (25.6)	35,415 (22.5)	
	60-69	8,217 (19:8)	25,416 (16.2)	
	70-79	2,523 (6.1)	9,083 (5.8)	
	80+	252 (0.6)	1,976 (1.3)	<0.001
Italian area	Northern	29,072 (70.1)	104,605 (66.5)	
	Central	8,282 (20.0)	32,600 (20.7)	
	Southern and islands	4,096 (9.9)	19,754 (12.6)	
	Abroad or unknown	23 (0.1)	390 (0.2)	<0.001
Educational level	Low	1,381 (3.3)	10,473 (6.7)	
	Middle	12,934 (31.2)	55,866 (35.5)	
	High	27,158 (65.5)	91,010 (57.8)	<0.001
Education	None	19 (0.0)	253 (0.2)	
	Elementary school	36 (0.1)	987 (0.6)	
	Secondary school license	1,326 (3.2)	9,233 (5.9)	
	Secondary school diploma	12,934 (31.2)	55,866 (35.5)	
	University degree	18,263 (44.0)	63,666 (40.5)	
	Postgraduate degree	8,895 (21.4)	27,344 (17.4)	<0.001
Employment status	Employed	29,299 (70.6)	107,557 (68.4)	
	Student	1,703 (4.1)	11,733 (7.5)	
	Unemployed	1,671 (4.0)	7,513 (4.8)	
	Retired	6,611 (15.9)	21,697 (13.8)	
	Other	2,189 (5.3)	8,849 (5.6)	<0.001
Healthcare workers	No	38,179 (92.1)	145,907 (92.7)	
	Yes	3,294 (7.9)	11,442 (7.3)	<0.001
N° of morbidities	None	25,466 (61.4)	101,791 (64.7)	
	One	11,628 (28.0)	40,736 (25.9)	
	Two	3,356 (8.1)	11,181 (7.1)	
	Three or more	1,023 (2.5)	3,641 (2.3)	<0.001
Number of rooms in the house	One	414 (1.0)	1,752 (1.1)	
	Two	3,998 (9.6)	15,583 (9.9)	
	Three	10,596 (25.5)	40,403 (25.7)	
	More than three	26,465 (63.8)	99,611 (63.3)	0.049
Number of cohabitant people	No one	5,713 (13.8)	19,383 (12.3)	
	One	13,819 (33.3)	49,205 (31.3)	
	Two	9,611 (23.2)	37,699 (24.0)	
	More than two	12,330 (29.7)	51,062 (32.5)	<0.001

Table S4. Standardized response rates per 100,000 inhabitants by Italian region over the phase II study period.

Region	Frequency	Percentage	Italian population*	Response rate
Abruzzo	330	0.8	1,311,580	25.2
Basilicata	110	0.3	562,869	19.5
Calabria	199	0.5	1,947,131	10.2
Campania	886	2.1	5,801,692	15.3
Emilia-Romagna	4,485	10.8	4,459,477	100.6
Friuli-Venezia Giulia	812	2.0	1,215,220	66.8
Lazio	4,072	9.8	5,879,082	69.3
Liguria	1494	3.6	1,550,640	96.3
Lombardia	13,832	33.4	10,057,370	137.5
Marche	710	1.7	1,525,271	46.5
Molise	63	0.2	305,617	20.6
Piemonte	4,644	11.2	4,356,406	106.6
Puglia	918	2.2	4,029,053	22.8
Sardegna	868	2.1	1,639,591	52.9
Sicilia	722	1.7	4,999,891	14.4
Toscana	3,188	7.7	3,729,641	85.5
Trentino	598	1.4	1,072,276	55.8
Umbria	312	0.8	882,015	35.4
Valle d'Aosta	76	0.2	125,666	60.5
Veneto	3,131	7.5	4,905,854	63.8

*Resident in Italy on 1 January 2020 (ISTAT)

Table S5. Frequency of self-reported symptoms by sex and by positivity to NPS and/or ST between March 2020 and February 2021 (*n*=41,473)

Self-reported symptoms	Total (*n*=41,473)	Total positive cases (*n*=4,411)	Females (*n*=25,146)	Females positive cases (*n*=2,716)	Males (*n*=16,327)	Males positive cases (*n*=1,695)
Fever	6,290 (15.2)	2,560 (58.0)	3,755 (14.9)	1,504 (55.4)	2,535 (15.5)	1,056 (62.3)
Cough	6,159 (14.9)	1,908 (43.3)	3,672 (14.6)	1,142 (42.1)	2,487 (14.9)	766 (45.2)
Headache	11,554 (27.9)	2,324 (52.7)	8,027 (31.9)	1,578 (58.1)	3,527 (21.6)	746 (44.0)
Myalgia	9,064 (21.9)	2,849 (64.6)	6,066 (24.1)	1,837 (67.6)	2,998 (18.4)	1,012 (59.7)
Shortness of breath	3,272 (7.9)	1,364 (30.9)	2,134 (8.5)	893 (32.9)	1,138 (7.0)	471 (27.8)
Chest pain	2,054 (5.0)	731 (16.6)	1,346 (5.4)	495 (18.2)	708 (4.3)	236 (13.9)
Gastrointestinal	7,330 (17.7)	1,553 (35.2)	4,840 (19.2)	1,047 (38.6)	2,490 (15.3)	506 (29.9)
Sore throat/rhinorrhoea	10,157 (24.5)	1,927 (43.7)	6,493 (25.8)	1,256 (46.2)	3,664 (22.4)	671 (39.6)
Dysgeusia	3,299 (8.0)	2,129 (48.3)	2,205 (8.8)	1,418 (52.2)	1,094 (6.7)	711 (42.0)
Anosmia	3,391 (8.2)	2,275 (51.6)	2,280 (9.1)	1,518 (55.9)	1,111 (6.8)	757 (44.7)
Loss of appetite	1,778 (4.3)	932 (21.1)	1,229 (4.9)	639 (23.5)	549 (3.4)	293 (17.3)
Cardiological	2,732 (6.6)	679 (15.4)	1,951 (7.8)	499 (18.4)	781 (4.8)	180 (10.6)
Dermatological	2,304 (25.6)	456 (10.3)	1,534 (6.1)	316 (11.6)	770 (4.7)	140 (8.3)
Neurological	1,747 (4.2)	648 (14.7)	1,285 (5.1)	490 (18.0)	462 (2.8)	158 (9.3)

Number are counts and row percentages
